# Live biotherapeutic products: the importance of a defined regulatory framework

**DOI:** 10.1038/s12276-020-0437-6

**Published:** 2020-09-10

**Authors:** Magali Cordaillat-Simmons, Alice Rouanet, Bruno Pot

**Affiliations:** 1 Pharmabiotic Research Institute (PRI), 1 Quai Vallière, Résidence l´île verte, 11100 Narbonne, France; 2 Research Group of Industrial Microbiology and Food Biotechnology (IMDO), Vrije Universiteit Brussel, Pleinlaan 2, G7, 1050 Elsene, Belgium

**Keywords:** Biological therapy, Biologics

## Abstract

Probiotics have been defined as “Live microorganisms that when administered in adequate amounts confer a health benefit on the host”. This definition covers a wide range of applications, target populations and (combinations of) microorganisms. Improved knowledge on the importance of the microbiota in terms of health and disease has further diversified the potential scope of a probiotic intervention, whether intended to reach the market as a food, a food supplement or a drug, depending on the intended use. However, the increased interest in the clinical application of probiotics may require specific attention given their administration in a diseased population. In addition to safety, the impact of the type of product, in terms of quality, production method and, e.g., the acceptance of side effects, is now part of the current regulatory constraints for developers. In the European Union, foods are regulated by the European Food Safety Authority and drugs by the European Medicines Agency; in the United States, the Food and Drug Administration (FDA) deals with both categories. More recently, the FDA has defined a new “live biotherapeutic products” (LBP) category, clarifying pharmaceutical expectations. Since 2019, the quality requirements for this category of drug products have also been clarified by the European Pharmacopoeia (Ph. Eur.). Similar to all products intended to prevent or treat diseases, LBPs will have to be registered as medicinal products to reach the market in the US and in Europe. In this area, regulatory authorities and the pharmaceutical industry will routinely use guidelines of the “International Council for Harmonization of Technical Requirements for Pharmaceuticals for Human Use” (ICH). Although ICH guidelines are not legally binding, they provide very important recommendations, recognized by almost all drug authorities in the world. In this review, we discuss some aspects of this regulatory framework, especially focusing on products with an intended use in a diseased or vulnerable target population.

## Prevention and treatment: When living microbes become medicines

Regulatory frameworks must be stretched to include microbe-based medicines such as “live biotherapeutic products” (LBPs). Some LBPs are similar by nature to probiotics, health-enhancing living microbes, except that they are meant to treat or prevent disease in sick or vulnerable individuals. Bruno Pot at the Vrije Universiteit Brussel in Belgium and co-workers have reviewed how current food and drug regulations in the United States and the European Union apply to living microorganisms. As for all drugs, in the case of LBPs, quality, safety, and efficacy must be demonstrated, but LBPs pose extra challenges because they contain live organisms, raising issues of stability over time and batch-to-batch variation. Differences in patients’ microbiomes and diets add further complications. This review helps to outline a way forward in this new and developing field of medicinal products.

## Introduction

Over the last few decades, biomedical science has evolved from a state where all microorganisms are considered health threats towards a better understanding of the importance of microorganisms in supporting and maintaining important physiological functions of the host. It is now commonly accepted that the microbiome is important for human health and that alterations in composition, relative abundances or constituents can lead to disease. To influence the microbiome with the intention to maintain or improve health, it is critical to better understand this microbiome–host relationship. Large-scale research programs such as Meta Hit^1^ or the Human Microbiome Project^2^ have allowed the identification of new strains and/or new microbial functions useful in the development of potential prophylactic or therapeutic applications.

Although this concept seems very recent, the idea to improve human health by impacting the gut microbiome is not novel, as in 1907, Metchnikoff already stated: “The dependence of the intestinal microbes on the food makes it possible to adopt measures to modify the flora in our bodies and to replace the harmful microbes by useful microbes”^3^. This concept was the basis for the early “probiotic” concept, much later officially defined by WHO/FAO in 2001^4^.

## How are “probiotics” regulated?

### Avoiding confusion between the product type and its regulatory status

Historically, two kinds of products, able to maintain or “rebalance” the microbiome, were developed. In the twentieth century, we saw the emergence of dietary supplements and foods with live microorganisms that were generally called “probiotics”, as well as registered medicines for which the active substance comprised living microbial strains and that were granted national marketing authorization in a number of European countries. Dietary supplements/foods were claiming *health* benefits, and the drug types were to have therapeutic or prophylactic activity in human diseases. However, no consensus existed on how to assess and demonstrate the benefits of live microorganisms, as, at that time, neither the food nor the drug competent authorities had clear rules or procedures for their evaluation.

Even if the borders between dietary supplements and drugs seems blurred, the regulatory difference is very clear: food supplements are intended to maintain or enhance a healthy state in a healthy or at-risk population; drugs are intended to cure or prevent a disease or pathophysiological state in unhealthy or diseased humans. This rule is independent of the nature of the product or its composition and is internationally accepted.

**Fig. 1:**
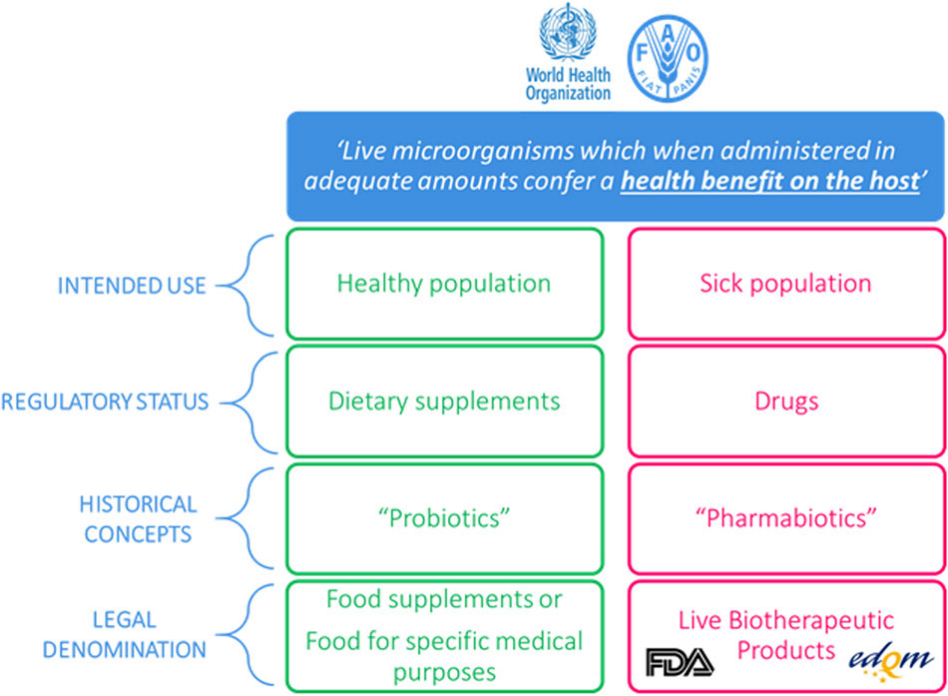
Clarification of the terminology and corresponding regulatory status.

In 2001, an FAO and WHO expert group defined “probiotics” as “Live microorganisms which when administered in adequate amounts confer a health benefit on the host”^4^. Regulatory speaking, this definition may be suitable to cover both a food or food supplement and a drug, depending on the intended use and the target population, as explained above. For the general public, the term probiotic, however, is most often related to a food or a food supplement and far less often to a drug or medicinal product. Therefore, given the abovementioned profound regulatory difference, it makes sense to consider a different naming for pharmaceutical products containing live microorganisms as active substances. Figure 1 illustrates this from a historical and regulatory perspective.

Product developers, when considering bringing a new product to the market, should therefore address the initial and crucial question of its intended use, independent of the composition of the product, allowing them to comply with the appropriate requirements stemming from the inherent regulatory status of the product.

### The early days of live biotherapeutic products

In an attempt to avoid confusion between the nature of the product and its regulatory status, the term “pharmabiotics” started to appear in the literature, addressing products containing live microorganisms with the intention to prevent or treat disease. However, this terminology had no legal ground and was not internationally recognized, mainly because it could include more than just living microorganisms.

In 2010, the Food and Drug Administration (FDA) was the first competent authority to propose to consider the drug status for these products with a first guideline draft^5^, in which they created a new term for products containing live microorganisms that were “applicable to the prevention, treatment, or cure of a disease or condition in human beings”. In 2012, this FDA guideline^5^ was published and officially created the LBP category. In 2019, with the publication of the Ph. Eur. Monograph^6^, the European Directorate for the Quality of Medicines and healthcare (EDQM) also officially accepted LBPs as a new category of medicinal products for the European market.

## Requirements for medicinal products with LBPs

The development of medicinal products has been harmonized since 1990 through the International Council for Harmonization of Technical Requirements for Pharmaceuticals for Human Use (ICH), which provides guidelines on the common expectations of the regulatory authorities (FDA in the US, European Medicines Agency (EMA) in the EU, and the Japanese Health Authority in Japan) and the pharmaceutical industry regarding drug registration. Although ICH guidelines are not “binding”, they make very important recommendations, recognized by almost all drug authorities in the world. They should be taken into account for new drug developments and registrations, as they address very important concepts in terms of the safety, effectiveness and quality of medicines worldwide.

To obtain marketing authorization, all new drugs need to demonstrate a positive benefit–risk balance, and, to that end, quality, safety and efficacy information must be documented according to a common format, i.e. the Common Technical Document (CTD). ICH guidelines assist developers in fulfilling all requirements expected by the drug authorities, including the required data and rationale to demonstrate this positive benefit–risk ratio.

**Fig. 2:**
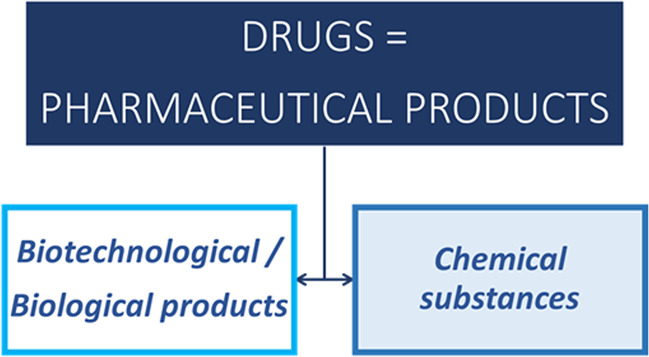
Different regulatory frameworks in ICH^7^ guidelines based on the nature of the medicinal product.

In all of the international drug guidelines, there is a clear distinction between “new chemical entities” (NCEs) and “biotechnological products” (Fig. 2^7^), and appropriate guidance exists for both categories. It is of course mandatory for all sponsors involved in the development of a new drug within one of these subcategories to demonstrate the quality, safety and efficacy of the product. However, there are specificities in the way to address the expectations, e.g., involving different methods for the characterization, demonstration of structure, batch definition, etc.

**Fig. 3:**
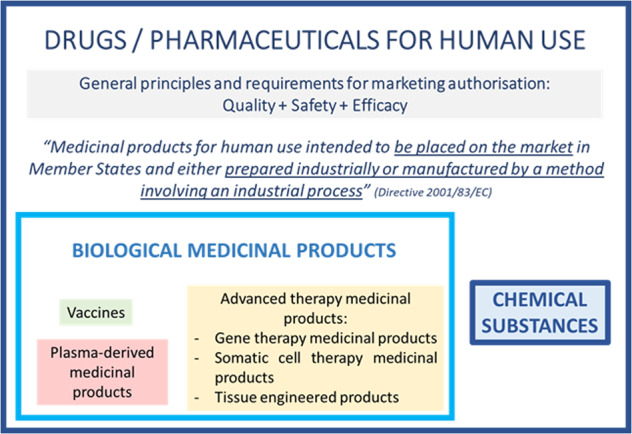
EU legislative framework relative to biological medicinal products.

The FDA as well as the EMA are also applying this dichotomy. Moreover, at the EU level, the legislator has defined some subcategories within the biological category, as illustrated in Fig. 3^8^.

In the European legislative framework, LBPs currently have no “separate status”. LBPs are by nature considered biological medicinal products as the active substances are live microorganisms, which are biological substances. As such, LBPs have to comply with the biological medicinal product legislative and regulatory framework. Therefore, in the absence of a specific LBP regulation, developers should rely on applicable and relevant regulatory concepts available for the subcategories of biological medicinal products, even if their LBPs are not perfectly within the scope of these specific legislations/guidelines (e.g., advanced-therapy medicinal products, cell-based therapies, etc.), as the spirit of several of these guidelines is often applicable and helpful for LBPs, as explained below.

### The benefit–risk ratio concept

The demonstration of a positive benefit–risk balance must be through quality, safety and efficacy data obtained from robust and validated nonclinical and clinical studies. For competent authorities, the benefit–risk ratio is the keystone of a drug evaluation dossier. The EMA’s reflection paper highlights the fact that “The assessment of the benefits and risks in the context of a new drug application is a central element of the scientific evaluation of a marketing authorisation application (…). The assessment must reach, as objectively as possible, a sufficient level of confidence that a set level of quality, efficacy and safety of the new medicinal product has been demonstrated. This requires evaluation of all relevant data as well as the use of judgement and arguments. (…) marketing authorisation shall be refused if the benefit–risk balance is not considered to be favourable or if therapeutic efficacy is insufficiently substantiated”^9^. The FDA equally stated: “Simply put, for a drug to be approved for marketing, FDA must determine that the drug is effective and that its expected benefits outweigh its potential risks to patients. This assessment is informed by an extensive body of evidence about the drug’s safety and efficacy, submitted by an applicant in a New Drug Application (NDA) or Biologics Licensing Application (BLA)”^10^.

Therefore, the challenge in developing an LBP or any other type of microbiome-based medicinal product is the documentation of the benefit–risk ratio with suitable and robust studies.

### Three important pillars in the demonstration of the benefit–risk balance

Regulatory guidelines precisely state which data are and what information is required by drug competent authorities. Some of them also recommend different design types for nonclinical and clinical studies^11^. Often, such proposed designs were developed from formerly granted marketing authorization dossiers and may not be totally relevant or appropriate for LBPs or, more globally, for “microbiotic medicinal products” (MMPs). Therefore, the challenge for sponsors developing these types of products lies in the difficulty of responding adequately to the regulatory requirements while proposing their most suitable, innovative designs for their nonclinical and clinical studies.

#### How can the quality of LBPs be ensured and demonstrated?

Quality parameters must be understood as key elements in drug assessments and should also be thoroughly addressed according to existing guidelines, such as the FDA guideline^5^ or the Ph. Eur. Monograph on LBPs^6^.

According to the FDA^12^, quality is built into pharmaceutical products by design through a comprehensive understanding of the following:

- The intended therapeutic objectives, the patient population, the route of administration, and the pharmacological, toxicological, and pharmacokinetic characteristics of a drug.
- The chemical, physical, and biopharmaceutical characteristics of a drug.
- The design of the product and the selection of product components and packaging on the basis of the drug attributes listed above.
- The design of the manufacturing processes, using principles of engineering, material science, and quality assurance to ensure acceptable and reproducible product quality and performance throughout the product’s shelf life.

The ICH Q11 regulatory guidance^13^ on drug substance manufacture and quality also establishes some general quality principles that are applicable to LBPs, e.g., stating that “the intended quality of the drug substance should be determined through consideration of its use in the drug product as well as from knowledge and understanding of its physical, chemical, biological, and microbiological properties or characteristics, which can influence the development of the drug product”. Indeed, a quality target product profile (QTPP) should be established, and all attributes identified as critical for the quality, i.e., critical quality attributes (CQAs), should be listed very early in development^14^. Knowledge and understanding of the CQAs as well as the level of information included in the QTPP may evolve during the course of development. In the case of LBPs, such an approach, derived from a “quality by design” mindset, is primordial, as the regulatory challenges faced by LBP sponsors will require more scientific reflection than conventional drugs due to the absence of specific guidelines for their nonclinical and clinical studies.

As for other drug products, strict good manufacturing practices (GMPs) always apply to the production of LBPs. In general, GMPs are directed towards the elimination of microorganisms from the end product and, in principle, should guarantee a contamination-free product. However, in the case of LBPs, the intention is quite opposite, namely, guaranteeing the viability of the desired microorganism(s) in the final product, at the end of the claimed shelf life and with no process variation, while avoiding contamination with other undesired microorganisms. GMP documentation covers procedures not only linked to purity but also to identity, stability and potency at all stages of the lifecycle and must be of high quality. GMP documentation should cover every activity, from the establishment of a master cell bank, the development of the processes for fermentation and biomass concentration, and the lyophilization process up to the production of the final product and the appropriate stability studies^15^.

It is a fact that the growth of live microorganisms is not easily controlled, neither at the level of production nor during the remaining part of the lifecycle. Factors that are generally considered important are the quality of the raw materials (which should be of pharmaceutical grade and preferably free of animal-derived substances), the manufacturing process (including the production facility, the environmental conditions and the equipment used), the hygiene and sanitation training of staff and the quality monitoring and control procedures. Some factors below are briefly discussed for their regulatory importance. It will almost certainly be time- and cost-saving to consider those aspects from the very early design phases of the product development cycle.

- Batch-to-batch variation for LBPs differs considerably from batch-to-batch variation in chemical manufacturing processes, with LBPs being known to represent a rather complex case. Specific parameters important for biologicals have been published by Jenzsch et al.^16^, who describe a number of parameters able to improve batch-to-batch reproducibility in microbial cultures, albeit focusing on recombinant protein production. The authors describe two main engineering challenges. As mentioned above, it is essential to define a robust operational procedure with the given constraints and expected variants following expected process fluctuations. In regulatory terms, it is important to “identify and describe the process attributes (critical parameters) that can influence batch reproducibility, product performance and drug product quality”^7^. Second, when executing this process, all other random fluctuations or disturbances must be eliminated by means of feedback control systems. Without these controls, the reproducibility of biomass cultivation might not be very high, a fact recognized by the FDA^16^, which advises developers to (i) define science-based principles and tools supporting manufacturing innovation and (ii) provide a strategy for regulatory implementation that will accommodate innovation, including tools for real-time automated process monitoring and control. Therefore, the early-stage modeling of possible process variations can have an important role in quality improvement, given that random fluctuations or disturbances are also efficiently addressed during the real manufacturing process. The quality-by-design approach allows such development and relies on the updating of the QTPP as well as the CQAs and their corresponding values (i.e., the specifications of the substances or products) along the history of the process development. The ICH is also advising an anticipation-based quality approach: “An enhanced approach to manufacturing process development generates better process and product understanding than the traditional approach, so sources of variability can be identified in a more systematic way. This allows for the development of more meaningful and efficient parametric, attribute, and procedural controls. The control strategy might be developed through several iterations as the level of process understanding increases during the product lifecycle”^13^. Limiting batch-to-batch variation without any doubt increases final product quality and therefore the chances of a positive evaluation of a dossier, as bias in the results due to variability in the product quality can be avoided.
- During the upscaling process, it is well known that the growth yields and strain characteristics of strains grown on a laboratory scale may differ from the yields and properties of strains grown on an industrial scale. For cost and pharmaceutical-quality requirement reasons, substrates used for final commercial production will most often be different from substrates used in the laboratory. The influence of such modification on the efficacy and safety of the product should clearly be evaluated and documented during development. Early understanding of the production constraints and the development of engineering batches containing pharma-grade raw materials (or raw materials of adequate quality) may allow developers to anticipate and avoid the repetition of proof-of-concept studies. Similarly, early identification of potency assays based on a general understanding of the mechanism(s) of action (MoA) may also allow developers to ensure that process evolution does not affect the efficacy and safety of the product. Clearly, it is important to anticipate process-related negative results in a timely manner before future, expensive, clinical trials.

A famous example of changes in the manufacturing process leading to significant differences in efficacy was described by Lebeer et al.^17^, who linked the adhesion properties of the strain *Lactobacillus rhamnosus* GG (LGG) to the presence of *spaCBA*-encoded pili at the surface of the strain by using a *spaCBA* pilus knockout mutant and a Caco-2 intestinal epithelial cell line. Later, the same group showed that these pili are susceptible to shearing stress, and when bacterial cells were subjected to centrifugal forces of 8000×*g*, the pili were damaged or lost, affecting the potency of the strain^18^. They concluded that it is important for industrial production of LGG that detrimental shearing stresses are avoided, as clinical efficacy may depend on the presence of intact pili. Another example was provided by Nivoliez et al.^19^ in a study were they comparatively assessed the cell wall characteristics of the strain *Lactobacillus rhamnosus* Lcr35, in which they could show that the manufacturing process influenced the cell wall properties. The authors concluded that the manufacturing process may affect efficacy and should therefore be taken into account early on during screening.

Therefore, it is of crucial importance for regulatory authorities to understand the production process for LBPs, including all constraints and possible variations, as well as the measures taken to avoid them and to improve reproducibility. Thorough documentation of the process development and justification of its evolution, as well as early identification of potency assays based on a global understanding of the MoA, will contribute to a better quality of the batches and a better reproducibility of the process. Therefore, the FDA guideline and the Ph. Eur. Monograph on LBPs, as well as the general biological drugs’ guidelines for quality^7,13^, should be analyzed very early during development to optimize the process development and quality controls, matching the regulator’s expectations and avoiding the replication of studies.

- Another important aspect for LBPs in regard to quality is the stability assessment. Stability is determined for the drug substance as well as the drug product and is necessary to anticipate product efficacy loss during shipping or storage, including phases between different production steps. The shelf life is defined based on the stability data to ensure that the dosage claimed efficacious is indeed present during the complete claimed shelf life. When different substrates for growth or finishing are used for the individual strains of a mixture, the mixing may change the final matrix composition, which may impact the viability of the composing strains and therefore the shelf life of the total product. Therefore, stability studies should be carried out for all intermediaries, drug substances and drug products in an LBP’s final formulation and packaging. Stability should be evaluated under different conditions relevant to the actual lifecycle of the product on the market (temperature, humidity, etc. of the countries of destination), covering shipping and storage, until the end of the claimed shelf life. Competent authorities are also sensitized to the documentation of the genetic stability of the strains themselves, within the cell banks and in the product. Genetic drift is often considered almost inexistent for stable strains; however, an assessment of the genetic stability of strains in banks is required, as such drift may lead to a loss of efficacy or even safety issues.

#### How can the safety of LBPs be demonstrated?

For a long time, because of their historical use in food products, microbial strains have been considered safe by nature. This safety concept has been officialized through the GRAS^20^ status by the FDA or the QPS concept^21^ by the EFSA based on the fact that the strains formerly used as ingredients of foods/dietary supplements have a long history of use in large human populations with no particular negative safety outcome. However, in regard to food legislation, the intended population is always a healthy population, and whatever the historical use of the strain, when such a strain is the active substance of a pharmaceutical product administered to humans for the prevention or treatment of a disease, safety demonstration in the targeted population is required.

Therefore, a strain with even a very long history of safe use in a healthy population cannot automatically be assigned the same level of safety in at-risk or debilitated patients. This is important, as, in contrast to what is sometimes described in earlier papers^22^, the regulatory status of a drug is not defined by the product or its nature, nor by its historical use. As noted above, it is defined solely by its intended use in the target population. In contrast to food products, medicinal products carry risks due to the weaknesses of their intended recipients. This implies that, according to the specific pathology, different risks and risk levels may need to be considered and assessed with regard to the expected benefit.

An EMA guideline on cell-based medicinal products reminds us that “at the beginning of the product development, an initial risk analysis may be performed based on existing knowledge of the type of product and its intended use”. “This should be updated by the applicant throughout the product lifecycle as data are collected to further characterize the risk. The comprehensive risk analysis should be used to justify the product development. It should also serve as a basis for the preparation of a risk management plan in accordance with the guideline on risk management systems for medicinal products for human use”^23^.

This concept is particularly important when developing safety and toxicity assessment programs for LBPs, as sponsors are dealing with at least three important interrelated paradigm shifts:

(i) The product itself does not reach the systemic circulation, although the results of its activities or metabolites may act directly or indirectly on (systemic) physiological functions of the host,
(ii) The toxicity is, therefore, not always directly related to the dosage, and
(iii) The translation of data from animals to humans is almost impossible due to the holobiont concept, a result of the coevolution of the microbiome and its human host that cannot be reproduced in animal species^24–27^.

Therefore, due to the biological nature of LBPs, preclinical development must be based on the assessment of the risks related to the microorganism(s), the product (final formulation) and the particular characteristics of the host and necessitates the use and even the development of suitable and innovative methods and models to assess such risks^28^. The understanding of the actual limits of these models is a prerequisite for the development of an acceptable preclinical program able to assess the safety and toxicity of LBPs. Therefore, product developers should engage in preclinical research programs that include combinations of in vitro and ex vivo models, organs on chips, and artificial organs, all the way up to in vivo models, to accumulate convincing, overlapping documentation that illustrates the global safety profile of their products adapted to the particular risks in the intended population. Although safety and toxicity studies are generally expected to be carried out on validated models, performed under GLP conditions, this aspect becomes problematic in the case of LBPs, as the novel models and methods may not currently be validated nor be at GLP level. Therefore, it is necessary to demonstrate the relevance of the approach taken and to negotiate about the suitability with the respective authorities on a case-by-case basis, as the approval or rejection of the preclinical and clinical trial results will finally reside with those agencies.

Overall, for LBPs, as well as for all biological drug products, safety must be considered from the very early steps of the development and always in relation to the target population and their clinical characteristics, as results might impact the early selection of the strain(s).

Finally, authorities will require developers to provide information on the origin of the strain. In terms of safety, information on the donor or the origin of the strain should be taken into account in the risk analysis, as some characteristics may influence the safety profile of the strain. Usually, strains are isolated from healthy individuals, but the documentation of the claimed “health status” is important for authorities. In line with this, all steps between the isolation and banking of the strains should also be documented, more particularly in the “manufacturing process development” part of the CTD (3.2.P.2.3)^7^, as raw materials used to grow and maintain the microorganisms, as well as the potential residuals, may also be part of the safety profile or influence the proof-of-concept demonstration for the strain(s).

#### How can the efficacy of LBPs be demonstrated?

Similar to preclinical studies, clinical studies with LBP have come with considerable concern. To date, the best documented sources are clinical trials with live microorganisms in the gastroenterology arena. Lessons learned from these were recently reviewed by Brüssow^29^. Given the strain and product specificity when considering microbiome-based products, conclusions from meta-analyses may be different from those found by individual, randomized controlled trials (RCTs), reflecting that specific products may not work under the specified conditions. These observations point towards the difficulties generally encountered in setting up clinical trials with live microorganisms. In the considerations below, the viability of the LBP is crucial and is different from the “postbiotic” concept that also might include intact but dead microorganisms.

From a regulatory perspective, clinical efficacy can theoretically only be considered proven if (i) confirmed by independent trials of acceptable quality (sufficiently powered, randomized and blinded, placebo-controlled trials), (ii) performed with a specified product including one or several active substance(s), reproducibly produced under GMP conditions, and (iii) applied to a well-defined patient population, using (iv) well-defined treatment conditions and dosage and (v) with preliminary defined, validated endpoint(s).

In practice, this turns out to be quite difficult for LBPs. The viability of microorganisms, even when produced under GMP conditions, can be affected by environmental factors (e.g., transport and storage conditions) as well as by host-related factors (e.g., health status, stomach pH, interference with diet, the composition of the recipient microbiota, ethnicity, etc.). These factors may also, or may not, have an impact on the potency or safety of the LBPs. In that sense, it seems that scientific reality may affect or conflict with regulatory requirements.

Indeed, LBPs directed towards the human gastrointestinal tract will not only be in competition with the indigenous microbiota but may also be impacted by the diet and, more largely, by environmental factors such as other medication, stress or cultural habits of patients. However, those potential biases should be identified and addressed in the design of the clinical trials, as they may influence the results. Preclinical work should already document the potential influence of the expected variation on the efficacy and safety of the products in the intended population to be able to anticipate and put in place the appropriate controls to avoid bias during clinical trials. Today, given the widely available omics technologies, the development of new LBPs can try to take into account the interaction of the LBP with the microbiome as well as with the diet. For many traditional drugs on the market, this type of information is not available, although diet and the microbiome could considerably affect drug efficacy, as recently discussed by Savage^30^.

As the intestinal microbiota is involved in the breakdown of many nonabsorbed dietary components, the composition of the microbiota as well as the diet may impact the host. Substrates from the diet, such as histidine, tyrosine, tryptophan, glutamate, dietary fiber or bile acids, will be converted to biologically active compounds, such as histamine, tyramine, serotonin, GABA, short-chain fatty acids or deoxycholic acid/lithocholic acid, which have roles in the maintenance of the intestinal epithelium; in the perception of pain, mood and behavior; in systemic immune, endocrinological and metabolic responses; and even in oncogenesis. The extensive metabolic capacity of the microbiota and its relatively high plasticity have been the basis for interest in the identification of dietary approaches able to steer and manipulate the bioconversion of food components from the diet^31^. (Epi)genetic mechanisms may even be involved. Perry et al.^32^ showed that the genomic copy number of salivary amylase is positively correlated with the amount of starch in the diet. This observation clearly illustrates that diet can act as an ecological driving force for the microbiota^33^, explaining why diet may also influence the effect of LBP administration, especially when applied for longer periods of time.

Although the relation between diet and microbiome composition is not always clear and still remains in its infancy^34^, there have been some attempts to support the fact that the microbiota may have a lifetime-long type of resilience, without ignoring individual, geographic and age-related differences^35^. According to Conlon and Bird^36^, diet does have a major role in shaping the composition and activity of the microbiota. Building on the well-studied, albeit less well-understood, effects of carbohydrates, it can be anticipated that taking into account the presence or absence of major macronutrients (dietary fats, proteins, …) may help to better understand some of the observed microbiota signatures.

Therefore, the conclusion might be that diet should be considered an environmental factor that has a potential influence on the performance of an LBP (in line with Schmidt et al.^34^). The impact of the diet may be more profound when the MoA relies on a particular metabolic activity rather than for a MoA that relies mainly on a direct immunological effect. More details on how intestinal microbes may also generate microbial metabolites that modulate mucosal and systemic immunity can be read in Ganesh and Versalovic^37^.

Another important regulatory aspect in regard to clinical assessment is the extrapolation of results between regions. Indeed, as the microbiome is highly influenced by the environment, the EMA position paper on this topic should be taken into account when designing clinical programs aimed at registration for a global market^38^. In this document, the EMA refers to the ICH E5 guideline on “*Ethnic Factors in the Acceptability of Foreign Clinical Data”*^39^. This guideline was created to facilitate the registration of medicinal products in different geographic regions. It recommends a framework for the evaluation of the impact of ethnic factors on the potency and safety of a drug at a specific dose and dose regime. The document is mainly intended for regulatory assessors, allowing them to evaluate how different factors may complicate the evaluation of foreign data in an EU perspective, but it is definitely also of interest to the industry when planning clinical trials in different geographical areas or considering an international or even global commercialization of a drug. An important conclusion from the document is that when “foreign clinical data” (data obtained outside the EU) do not meet the European regulatory requirements, regulatory authorities may require additional clinical tests, such as

- Clinical studies in different subsets of the population, such as patients with specific deficiencies or diseases,
- Clinical studies using different comparators at the new region’s approved dosage and dose regimen, and
- Drug-drug interaction studies.

In general, however, “when the regulatory authority of the new region is presented with a clinical data package that fulfils its regulatory requirements, the authority should request only those additional data necessary to assess the ability to extrapolate foreign data from the Complete Clinical Data Package to the new region”^39^.

In the case of LBPs directed to the gut microbiota, sensitivity to ethnic factors may, among others, be reflected in differences in the general diet. Therefore, regulatory authorities might ask about the available data. In most cases, a single “bridging” trial confirming the ability to extrapolate data from the original region/diet to the new region/diet should suffice, and no full-scaled replication study should be needed.

The complexity of this microbiota-host interaction (the holobiont theory) and the multiplicity of the potential MoA of the LBP concerned might further blur a readout based on a single biomarker. As an example, the intake of an LBP intended to influence the host’s immune system may have a secondary impact via cross feeding or through microbiological interactions that might blur or amplify the envisaged effect. Therefore, it would be advised to have some prior knowledge on the MoA of the product, preferably under different conditions. The importance of preclinical studies in this case cannot be underestimated.

In the field of LBPs, straightforward biomarkers, such as cholesterol for cardiovascular health, are very scarce. Again, fundamental knowledge of the MoA may be helpful but may be prone to numerous lateral influences and/or depend on the target population and therapeutic targets. According to the NIH^40^, the application fields for living microorganisms are already very broad, and they are depicted in Table 1.

**Table 1:** Modified list of NIH-mentioned application fields for LBPs.

Gastrointestinal conditions	Antibiotic-associated diarrhea *Clostridioides difficile* infection Constipation Diarrhea caused by cancer treatment Diverticular disease Inflammatory bowel disease Irritable bowel syndrome Traveler’s diarrhea
Conditions in infants	Infant colic Necrotizing enterocolitis Sepsis in infants
Dental disorders	Dental caries (tooth decay) Periodontal diseases (gum disease)
Conditions related to allergy	Allergic Rhinitis (hay fever) Asthma Atopic dermatitis Prevention of allergies
Gut–brain axis related conditions	Anxiety and stress Cognition and cognitive reactivity Depression and mood Autism spectrum disorder Schizophrenia Parkinson disease Alzheimer disease
Other conditions	Acne Hepatic encephalopathy Upper respiratory infections Urinary tract infections Genital tract disorders

Obviously, reliable biomarkers may not be available for all these types of interventions. Over the last year, however, the increased number of reports on microbiota-related interventions has resulted in a large number of disease markers that have been impacted directly or indirectly by the respective interventions (Table 2)^41–46^. It is important to discriminate between general disease-related biomarkers and biomarkers for the microbiota. Critical to both is the level of validation. Many disease-related markers have been validated in the past. Their use in microbiota-related interventions is perfectly defendable, and their outcomes will be considered valid if obtained by standard clinical research practices. Microbiota-related biomarkers are often not validated. Although their use may be more restricted in terms of regulatory acceptance, there are a number of documents that can help to set up proper validation studies^47^. It can be expected that as research efforts in the microbiome field will further develop and expand, validation efforts for microbiome-related markers will become more available.

**Table 2:** Examples of biomarkers recently used in specific disease settings.

Application	Markers	Refs.
Inflammation, oxidative stress and pregnancy outcomes in gestational diabetes	Fasting plasma glucose (FPG) Serum high-sensitivity C-reactive protein (hs-CRP) Plasma malondialdehyde (MDA) concentrations MDA/TAC ratio Significant increase in total antioxidant capacity (TAC) levels	38
Inflammatory disorders, including allergies, diabetes, obesity, heart diseases and cancer	Global cytokines MicroRNA gene expression analyses	39
Aflatoxin exposure	Aflatoxin B(1)-N(7)-guanine	40
Oxidative stress in diabetic patients	Malondialdehyde [MDA] Glutathione [GSH] Nitric oxide [NO] Total antioxidant capacity [TAC]	41
MS patients	Malondialdehyde 8-Hydroxy-2′-deoxyguanosine IL-6 C-reactive protein IL-10, nitric oxide	42
Parkinson disease	Humans alpha synuclein accumulation Dopaminergic loss in the substantia nigra pars compacta Synucleinopathy Lewy bodies	43

## Conclusion

Regulatory and market success for an LBP will depend on the quality of the development involving the credible demonstration of safety and efficacy in the intended population. Development strategies should therefore be decided as early as possible in addition to the main commercial key factors. Currently, there exists no standard clinical trial format, as products, target populations and application modes are likely to differ on a case-by-case basis.

The registration of LBPs as drugs requires that the developers comply with a set of regulatory requirements that in the EU are not specifically defined for LBPs at this moment, even if they were partly laid out by the FDA since 2012^5^. Therefore, developers should use the possibilities offered by regulatory authorities to bilaterally discuss all critical steps of their product development. We advise using this period in full before submitting the final dossier, avoiding errors in the procedure or gaps in the data provided that may invalidate a costly dossier or, in the best case, lead to additional time-consuming research activities. Clinicians should preferably also be involved in contact with authorities, as they may have a more realistic appreciation of the feasibility/reliability of the proposed steps in the intended population.

In the absence of a more generic procedure, developers may need to walk down more than one path. LBPs are not different from other biological products, and their development should comply with the spirit of the authorities’ expectations laid out in guidelines developed for these products. Different types of data on safety, toxicity or pharmacology related to the product, manufacturing or distribution, may apply. When a product is intended for international distribution, it is important to consider the different conditions from the start and, whenever possible, to choose the option that offers acceptable data for the different legal requirements.

Overall, the aim of any drug development, LBPs included, is the documentation and demonstration of quality, safety and efficacy. Such demonstration is framed by the characteristics and risks of the intended population, as well as the characteristics and risks of the strains and product components, so that the global benefit–risk ratio can be assessed regarding their intended use.
